# Rice Ear Counting Based on Image Segmentation and Establishment of a Dataset

**DOI:** 10.3390/plants10081625

**Published:** 2021-08-06

**Authors:** Hongmin Shao, Rong Tang, Yujie Lei, Jiong Mu, Yan Guan, Ying Xiang

**Affiliations:** 1 College of Information Engineering, Sichuan Agricultural University, Ya’an 625000, China; shaohongmin@stu.sicau.edu.cn (H.S.); tangrong@stu.sicau.edu.cn (R.T.); leiyujie@stu.sicau.edu.cn (Y.L.); guanyan@stu.sicau.edu.cn (Y.G.); xiangying@stu.sicau.edu.cn (Y.X.); 2 Sichuan Key Laboratory of Agricultural Information Engineering, Ya’an 625000, China

**Keywords:** deep learning, detection and counting, instance segmentation, agricultural automation

## Abstract

The real-time detection and counting of rice ears in fields is one of the most important methods for estimating rice yield. The traditional manual counting method has many disadvantages: it is time-consuming, inefficient and subjective. Therefore, the use of computer vision technology can improve the accuracy and efficiency of rice ear counting in the field. The contributions of this article are as follows. (1) This paper establishes a dataset containing 3300 rice ear samples, which represent various complex situations, including variable light and complex backgrounds, overlapping rice and overlapping leaves. The collected images were manually labeled, and a data enhancement method was used to increase the sample size. (2) This paper proposes a method that combines the LC-FCN (localization-based counting fully convolutional neural network) model based on transfer learning with the watershed algorithm for the recognition of dense rice images. The results show that the model is superior to traditional machine learning methods and the single-shot multibox detector (SSD) algorithm for target detection. Moreover, it is currently considered an advanced and innovative rice ear counting model. The mean absolute error (MAE) of the model on the 300-size test set is 2.99. The model can be used to calculate the number of rice ears in the field. In addition, it can provide reliable basic data for rice yield estimation and a rice dataset for research.

## 1. Introduction

Precision agriculture is an important trend in future agricultural development, among which agricultural informatization is a development direction vigorously advocated at present. The informatization of the agricultural industry will contribute to the intellectualization of agricultural management, improve the output of agricultural products and increase the economic benefits of the industry [1]. Agricultural informatization also plays an important role in the forecasting of agricultural product output, which helps to manage the agricultural product production cycle [2,3]. Deep learning technology is one of the contributors to promoting the development of agricultural informatization and intelligence, and is also widely used in various agriculture-related fields [4].

Deep learning (DL) is an important development direction and research hotspot of AI (artificial intelligence) technology. The basic principle is that the machine is based on complex algorithms and can independently analyze and master its laws or levels after learning from a large number of samples. The machine can automatically recognize targets, accurately classify data, or make predictions. Agricultural production has the characteristics of huge data, multiple impact factors, complex model mechanisms and continuous updating of empirical knowledge. DL can solve a large number of nonlinear problems in agriculture, after learning through neural networks. Therefore, in recent years, DL has been widely studied and applied in the agricultural field [5]. For example, in a botany study conducted in China, Zhang et al. [6] improved the typical deep confidence network model to identify diseases and insect pests of winter jujubes in greenhouses. In 2018, An et al. [7] identified four diseases of alfalfa by establishing a support vector machine (SVM) model. In the same year, Wang et al. [8] proposed a prediction model for cotton diseases and insect pests based on a self-adaptive discrimination deep belief network (DBN).

Rice is one of the most important food crops in the world. Nearly half of the world’s population feed on rice as a staple food. It is also China’s main food crop and an important economic crop, and sustainably increasing production has always been the primary goal of rice breeding research. With the rapid growth in China’s population, the consumption of rice is also steadily increasing, and the current discrepancy of more people and less land is still difficult to resolve in a short time. Therefore, the selection and breeding of high-yield rice has become key in field rice research. Traditional methods of measuring rice yield in the field are mostly destructive; that is, after the rice is harvested, a series of steps such as threshing, cleaning, drying and weighing are carried out, and the final yield is then determined. This method is time-consuming and labor-intensive, and it is easy to introduce artificial errors due to operational errors. Rice ear is an important vegetative reproductive organ of rice. The phenotypic traits, such as the ear length and grain number of rice ear, are closely related to the final yield. Therefore, it is an indicator of rice phenotypic parameters that can directly reflect the level of rice breeding [9].

This study explores the application of deep learning algorithm in rice crops using the related agricultural deep learning algorithm as a basis. One of the technical advantages of deep learning is its strong learning ability and suitability for massive data, meaning it can be used for detection and identification within a large sample of rice data. At present, numerous studies have been conducted on the direction of rice counting in deep learning. Duan et al. [10] proposed a neural network based on a deep convolutional encoder–decoder architecture for scene segmentation to achieve semantic segmentation of on-site rice ears. However, because this network requires two steps—offline training and online segmentation of rice images—it is not convenient enough. Tri et al. [11] used a combination of drone shooting and deep learning to predict the yield of rice fields, whereby drones were first used to perform image acquisition of rice fields. The classification of rice was then completed through deep learning models. For the estimation of rice yield, Tri et al. [11] manually counted one square meter of rice. After obtaining a large amount of data, the average rice yield per square meter was obtained. The averaged data were then applied to the entire paddy field. However, the prediction method based on sampling did not make predictions for each individual paddy field. Therefore, this method is prone to large errors due to different land environments.

With the rapid development of remote sensing, global satellite positioning system, microcomputer and other technologies, the remote sensing technology platform of micro-unmanned aerial vehicles has made great progress, providing technical support for the further development of precision agriculture. The information acquisition technology of micro-UAV has the characteristics of easy platform construction, low operation and maintenance cost, small size, light weight, simple operation, high flexibility and short operation cycle. It can make up for the shortcomings of the existing aerospace, aviation remote sensing and ground remote sensing systems and improve the ground crop monitoring system [12]. In particular, for small and medium-scale agricultural remote sensing applications, it can play a greater role and obtain more accurate agricultural information, which is of great significance to the development and application of crop information monitoring technology. UAVs equipped with mobile laser scanners, CCD cameras, spectrometers and thermal imaging cameras can not only record geometric profile data but also collect image information, laser backscatter intensity and hyperspectral and thermal information data [13,14]. UAVs can collect data repeatedly at relatively low cost, giving them unique advantages in multitemporal data collection. For example, using the advantages of UAVs in fine geometric measurement to finely monitor the growth height and distribution density of crops, the growth and development of crops can be precisely monitored, and the precision of crop monitoring can be improved. For example, the use of UAV hyperspectral image analysis technology to obtain soil fertility and crop diseases and insect pests can be used to guide variable fertilization and pesticide application to improve the use efficiency of chemical fertilizers and pesticides [15]. UAVs effectively make up for the limitations of traditional satellite remote sensing in data acquisition [12].

Similarly to Reza et al. [16], we chose to formulate statistics on the number of rice ears to more accurately estimate yield data. Reza et al. used K-means, which is a common classification method in machine learning, to classify and segment rice by graph cuts. However, the accuracy of this method is not high, and its relative error is 6–33%. In order to solve the above problems, this research applies a new concept of deep learning, namely a point-supervised convolutional neural network, to rice ear statistics. Combining the idea of point supervision with image segmentation, the rice ear counting model of the convolutional neural network is obtained after training. Then, it is tested on a 300-size test set. The comparison between the machine learning method and the object detection algorithm shows the usability of the proposed method.

This paper is divided into four sections. Section 1 introduces the significance and objectives of the identification research of rice ear. Section 2 introduces the process of image data collection and preprocessing, including image acquisition, image preprocessing and dataset creation. Then, we explain the algorithm model, the key method used in this study, which combines the semantic segmentation network algorithm and segmentation method. Section 3 presents the related experiments and the discussion of the experimental results. Finally, Section 4 discusses the conclusions and future prospects.

## 2. Materials and Methods

### 2.1. Experiment Field and Data Acquisition

**Figure 1:**
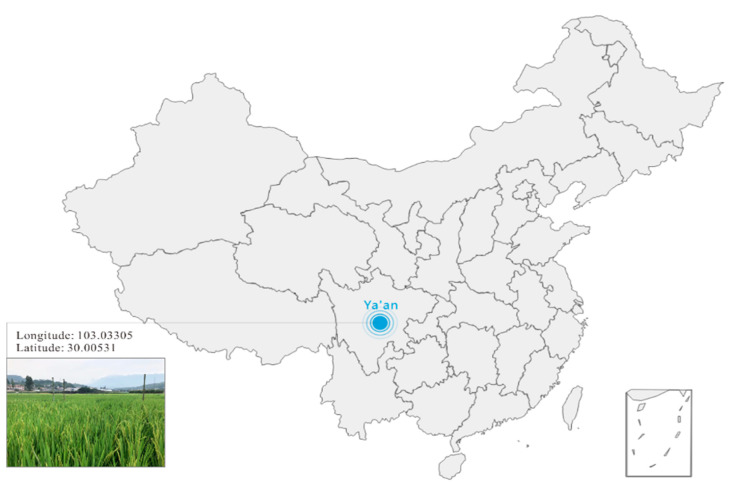
Location of the experimental area in Ya’an, as marked on the map.

The experimental area is located in the rice experimental field of Sichuan Agricultural University, Ya’an City, Sichuan Province, China, as shown in Figure 1. These photos were taken in cloudy weather at the end of July. The collection time was from 7:30 a.m. to 11:30 a.m. In order to ensure the stability and consistency of the experimental picture pixels, we made a square red paper frame with an inner frame of 50 cm and an outer frame of 80 cm. The shooting range was fixed with a paper frame to ensure imaging of rice in the same unit area, which avoided differences in quantity. This project used a lightweight fixed-wing drone with a weight of 840 g. Meanwhile, the drone was equipped with a lightweight camera. The camera was XTU S2 with 20MP camera pixels and its weight of only 75 g with SuperSmooth antishake, the image sensor was SONY IMX458 and data collection was performed at a shooting height of 3 m. A total of 1679 photos of rice were taken, including many different varieties with different growth cycles. The lighting conditions varied during shooting.

### 2.2. Image Preprocessing

**Figure 2:**
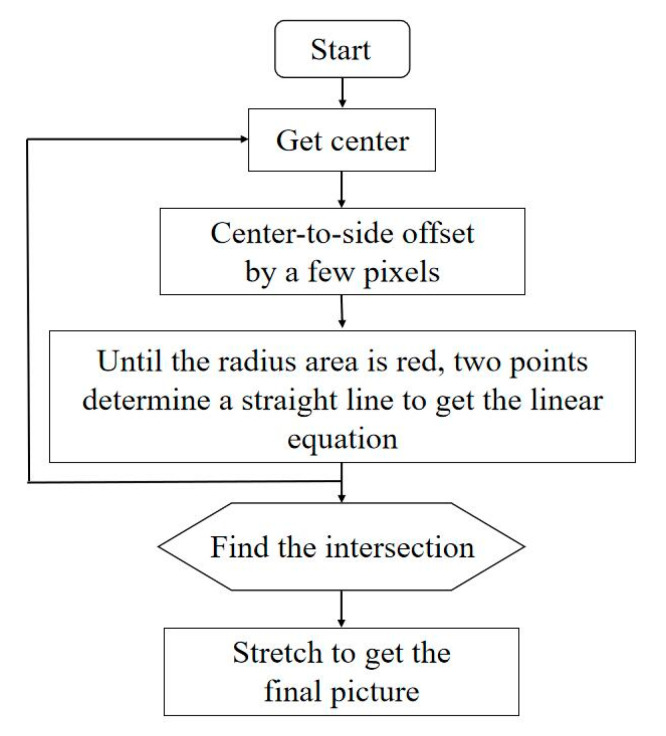
Preprocessing of original picture.

The image first needed to be trimmed, considering that only the rice within the red frame in the original rice image was needed in image preprocessing. The part inside the red frame was reserved, and the red frame and the area outside the red frame needed to be discarded. As shown in Figure 2, the processing included the following steps: (1) capture the center of the picture; (2) shift some pixels from the center to both sides to emit rays; (3) continue to explore until a certain radius area is red, then determine one straight line between two points to obtain the linear equation. After repeating the above three steps, find the intersection point, mark the graphic and then directly stretch it to obtain the final picture. After screening and sorting out the quality of photos, 1661 photos were obtained, and all the data were divided into three levels, as shown in Table 1.

### 2.3. Dataset Creation

**Figure 3:**
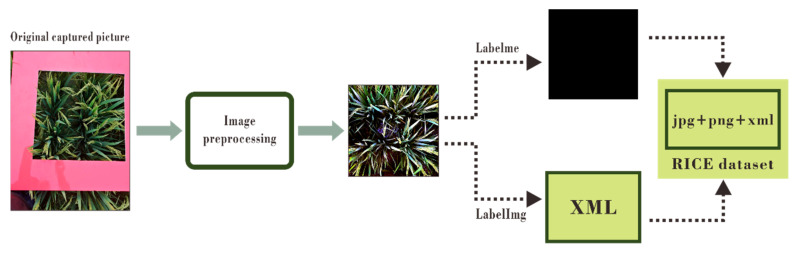
The complete process of dataset production.

Before labeling, all pictures were uniformly resized to approximately 25% of the original size. We used the LabelMe annotation tool (http://labelme.csail.mit.edu/Release3.0/ accessed on 12 April 2021) for point labeling. The rice ear was a one-pixel red dot, and the rest of the picture was the background area, which was all marked as black. In the end, a total of 1100 pictures were labeled. However, the neural networks required a larger number of samples to increase the model robustness, and the labeling process was complicated. Thus, we used data augmentation to increase the number of samples to 3300. Finally, we obtained a complete point annotation dataset and named it the RICE dataset. We divided the dataset into a training set and a test set at a ratio of 7:3. This dataset contained original pictures in JPG format and PNG annotation files, which can be used by other researchers studying point-supervised neural networks. In this research, we also needed to use the object detection algorithm to conduct comparative experiments. Therefore, we extracted a part of the pictures and used LabelImg (https://pypi.org/project/labelImg/ accessed on 12 April 2021) to label 100 pictures to form a small object detection dataset, which can also be used by researchers who study object detection. The complete production process of the dataset is shown in Figure 3.

### 2.4. LC-FCN Algorithm

**Figure 4:**
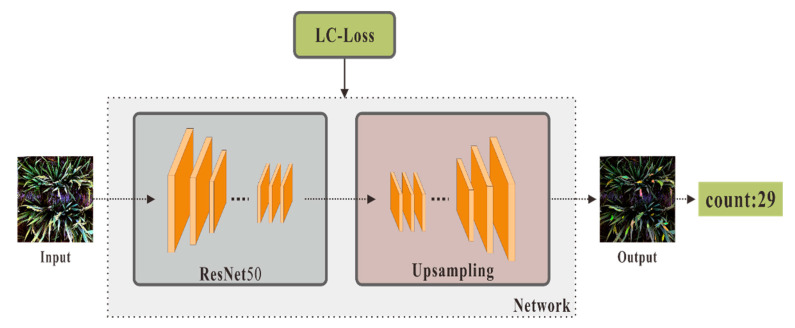
The overall processing flow of the LC-FCN network.

LC-FCN is a model proposed by H. Laradji [17] for detecting and counting cars on the road. It uses a combination of semantic segmentation network and instance segmentation algorithm. The traditional semantic segmentation method requires accurate prediction of the object shape. By contrast, the method for increasing LC loss does not require prediction of the exact shape; it only needs prediction of a small blob in an object. Such a method is also applicable to rice in a field environment. The object detection method has difficulty in accurately identifying various scattered and overlapping rice crops. The algorithm model of LC-FCN was selected to train the rice dataset on the basis of the advantages of LC-FCN. The detailed training process is described in detail below and is also presented in Figure 4.

In the first step, the input rice picture is subjected to the semantic segmentation of the rice through a residual network to obtain a feature map. In the second step, the feature map is upsampled to obtain an output picture consistent with the size of the original picture. Each pixel corresponds to the probability of belonging to the rice class. In the third step, the pixel is predicted to be a rice class and is set to 1. Otherwise, it is set to 0 to obtain the binary mask of the rice. Lastly, by finding the connected domains of the rice class, the predicted number of rice ears is acquired. A detailed description of the backbone network and the loss function is provided below.

ResFCN: FCN [18] is a pixel-level semantic segmentation network. Compared with CNN, it solves the classification problem at the pixel level and replaces the fully connected layer in the CNN with a fully convolutional layer. It can accept images of any size and outputs images of the same size. The backbone of the FCN used in this study is ResNet50 [19]. This network uses the idea of adding residual learning to the traditional convolutional neural network to solve gradient dispersion and accuracy degradation in deep networks. In the training of deep networks, both accuracy and speed can be guaranteed.

LC-Loss: In this study, we used the LC-Loss function based on localized counting loss. After providing the region blobs of each object, the model counted the number of regions. Given the particularity of the loss function, our surveillance signal should only be the position point of each object, not the bounding box. The counting loss based on positioning has four types: (1) image-level loss, (2) point-level loss, (3) split-loss, and (4) false-positive loss, as shown in Formula (1):

(1) $$L\left(S,T\right)=L_{I}\left(S,T\right)+L_{P}\left(S,T\right)+L_{S}\left(S,T\right)+L_{F}\left(S,T\right)$$

The specific explanation for Formula (1) is as follows. *T* represents the truth on the ground, and *S* represents the output of the network. Each pixel has a soft-max vector, which represents the probability that the pixel belongs to class C. Finally, the area is divided by taking the argmax of each pixel of the output. The four losses of the LC-Loss function are explained in more detail below.

Image-level loss: This type represents the image-level loss, which involves finding all semantic categories that exist in the original image. For the rice dataset, all the categories in the picture are determined, including the rice ears and the background. $C_{e}$ is the set of objects existing in the original picture. $C_{\neg e}$ represents the category and combination of nonexisting objects, and *S* represents the maximum probability that each pixel of *S* belongs to class C. The calculation process is as follows:

(2) $$L_{I}\left(S,T\right)=-\frac{1}{\left|C_{e}\right|}\underset{c\in C_{e}}{\sum }\log\left(S_{t_{c}c}\right)-\frac{1}{\left|C_{\neg e}\right|}\underset{c\in C_{\neg e}}{\sum }\log\left(1-S_{t_{c}c}\right)$$

Point-level loss: This type of loss encourages the model to correctly label a small group of supervised pixels in ground truth. It represents the supervised point set in ground truth. It also represents the position information of the object in the picture. Moreover, this item ignores all unlabeled points. It is calculated as follows:

(3) $$L_{P}\left(S,T\right)=-\underset{i\epsilon I_{S}}{\sum }\log\left(S_{iT_{i}}\right)$$

Split-loss: This type is used to split blobs. It can calculate the boundaries of objects based on their annotations and divide these boundaries into background classes. Formula (4) shows its calculation. Two methods are typically used to determine the boundary, namely the line segmentation method and the watershed segmentation method. In this study, we use the watershed segmentation method to determine the boundary.

(4) $$L_{F}\left(S,T\right)=\frac{1}{\left|E\right|}\underset{i\in E}{\sum }S_{i0}$$

False-positive loss: This loss suppression model outputs blobs that do not contain objects. By calculating the cost loss of all regions that do not include markers, regions other than rice ears that need to be predicted can be discarded. This process is critical to improving the effect of object counting. This model is calculated as follows:

(5) $$L_{F}\left(S,T\right)=-\underset{i\in B_{fp}}{\sum }\log S_{i0}$$

### 2.5. Implementation and Evaluation Index

The counting model in this study was trained, verified and tested on NVIDIA 2080. The parameters during training refer to the parameters in the original LC-FCN model. The batch size was set to 1, the momentum of the model was set to 0.9, and the weight decay was set to 0.0005. The pretrained model was trained at a learning rate of 0.00001. For better analysis, 180 epochs were trained. In this study, a trained model was used to perform counting tests on the rice dataset to verify the performance of the algorithm. The evaluation criteria had the following indicators.

Mean absolute error (MAE): The MAE is the average of the absolute errors, which can better reflect the actual situation of the predicted value errors. The main evaluation index used in this study was MAE.

(6) $$\mathrm{MAE}=\frac{1}{m}\overset{m}{\underset{i=1}{\sum }}\left|h\left(x_{i}\right)-y_{i}\right|$$

Root mean square error (RMSE): The RMSE measures the deviation between observed and true values. It is often used as a measure of the prediction results of machine learning models.

(7) $$\mathrm{RMSE}=\sqrt{\frac{1}{m}\overset{m}{\underset{i=1}{\sum }}{\left(h\left(x_{i}\right)-y_{i}\right)}^{2}}$$

Normalized root mean square error (nRMSE): The nRMSE is the RMSE divided by the range of observed values of a variable being predicted. The value is often expressed as a percentage, where lower values indicate less residual variance.

(8) $$\mathrm{nRMSE}=\frac{\mathrm{RMSE}}{y_{max}-y_{min}}$$

Accuracy: The accuracy calculation formula is as follows below. Accuracy calculates the ratio of the actual predicted number to the true number, and only the numerical accuracy is provided. Given the large difference in the number of rice ears in each image, the accuracy used in this study was only for reference.

(9) $$\mathrm{Acc}\_\mathrm{rate}=\overset{m}{\underset{i=1}{\sum }}\left(1-\frac{\left|h\left(x_{i}\right)-y_{i}\right|}{y_{i}}\right)$$

## 3. Results

### 3.1. Data Augmentation

**Figure 5:**
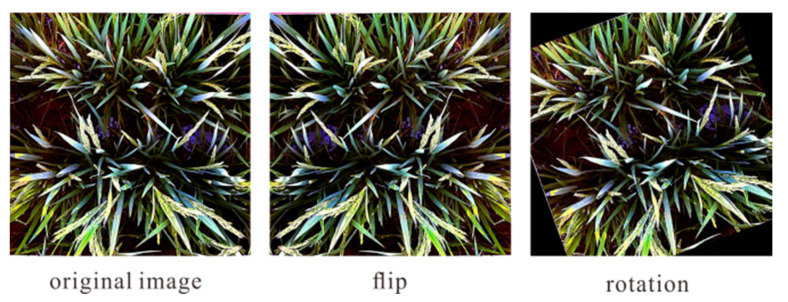
The original picture in the dataset and the picture after data enhancement.

**Figure 6:**
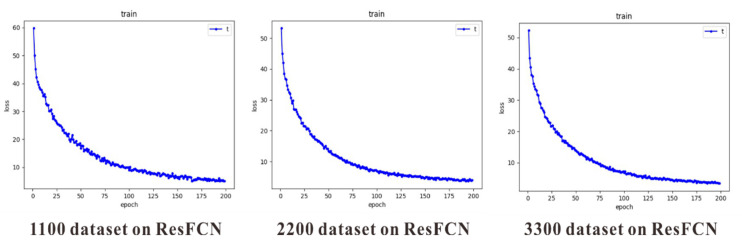
Training comparison of datasets of different sizes on same network after data augmentation.

**Figure 7:**
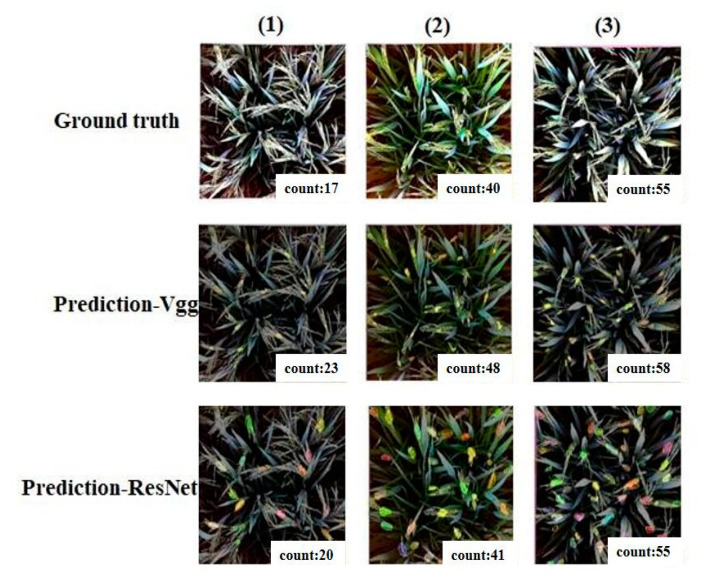
Test examples of ResNet and Vgg.

Data augmentation allows the use of limited data to generate additional data. Moreover, it increases the number of training samples and the diversity of multiple noises and improves the model robustness. This study used two data augmentation methods, including flip and rotation, to increase the 1100 sample size of the original dataset to 2200 and 3300 sample sizes, respectively. Figure 5 shows the original picture and the picture after data augmentation. When the three datasets of different sizes were input into the same network structure for training, the training situation became evidently different. Figure 6 is a comparison of datasets of different sizes trained on the same network. The larger the dataset, the smoother the loss curve, thus indicating that the training was more stable. The test results further verify that a larger dataset entails a better model, as shown in Table 2. No matter how the basic network changed, MAE decreased as the dataset increased on the same network. As a result, the dataset after data augmentation was added and the counting accuracy was significantly improved. The best MAE reached 2.99, RMSE could reach 5.40, nRMSE could become 7.01%, and the accuracy rate could reach 89.88%. The sampling test image is shown in Figure 7. This model can accomplish the goal of counting rice ears in a given area and serves as a basis for research in image-based counting of rice ears.

### 3.2. Comparison of Different Backbones

This study used two different basic network structures, namely Vgg-16 [20] and ResNet50. Vgg-16 is the most classic classification network. ResNet is based on Vgg and adds residual ideas. As shown in Table 3, rice models trained with ResFCN had higher accuracy. The same 1100 sample size dataset was trained. Then, the resulting model was input into the test set for comparison. The sample images are shown in Figure 7. The segmentation block trained by Vgg was larger, and the adhesions still remained. The rice blobs segmented by ResNet produced more accurate results. Therefore, the basic network of ResNet was more suitable for this study.

### 3.3. Comparison with Machine Learning

**Figure 8:**
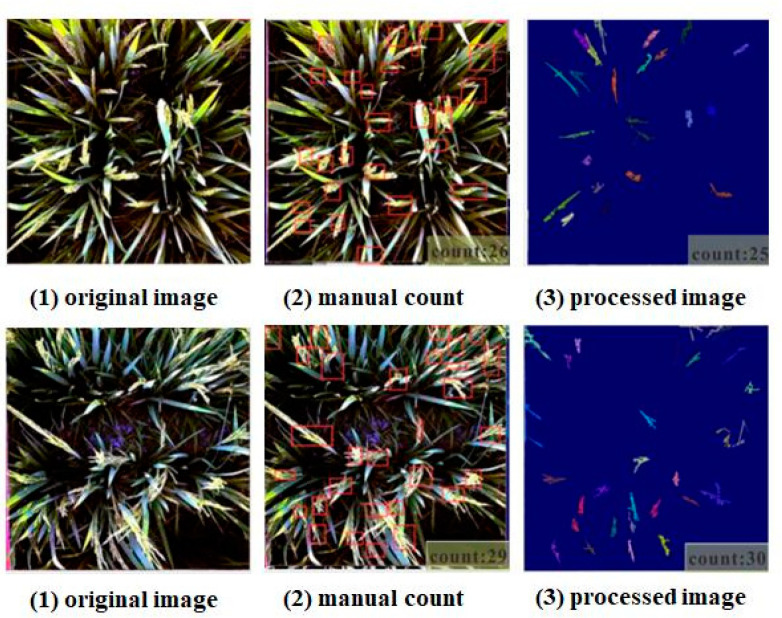
Result after using the traditional method of image processing.

This study also uses traditional image processing methods for rice counting to verify the practicability of this method. The image processing steps are as follows: (1) convert RGB format to HIS format; (2) extract the I component and binarize it; (3) use two steps to find the connected domain and remove the dirt and leaf noise; (4) remove the ear with the expansion through corrosion and obtain the picture of the leaf; (5) use the XOR (exclusive OR) operation on the results of steps 3 and 4 to obtain a picture of rice ears; (6) find the value of the connection and remove the other leaves; (7) obtain the final result. Five pictures were selected for processing and prediction, and MAE of 1.2. Two test pictures were selected, as shown in the figure. The test results of the two sampled pictures are shown in Figure 8. The accuracy was relatively high in terms of these five sample sizes. However, the mutual offset between the wrong number of rice ears and the missing number of rice ears was neglected. In practical applications, the efficiency of traditional machine learning methods is low, and it is difficult to use a batch of input images to output predictions. In comparison, the end-to-end method adopted in this study was simpler and faster.

### 3.4. With Object Detection

**Figure 9:**
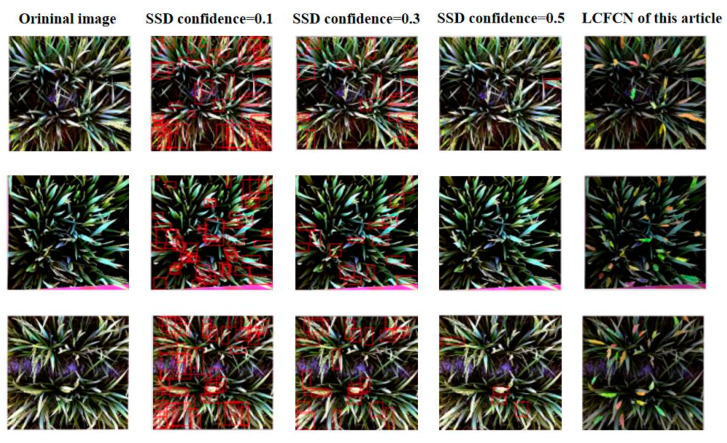
Comparison of three sets of test sample images. The first column is the original image. The second, third and fourth columns are the test results of the SSD method with confidence settings of 0.1, 0.3 and 0.5, respectively. The fifth column is the test results of the method in this study.

The method in this study uses image segmentation. In addition, computer vision has target detection classes. Therefore, it uses a more advanced SSD [21] algorithm than the method used in this study. In the final test results of SSD, MAE is 13.62, RMSE is 15.79, and the threshold is only set to 0.1. The test results are shown in Figure 9. When the threshold was set to 0.5, few large rice ears in the picture could be identified. When the threshold was set to 0.3, unobstructed and clear ears of rice could be identified. When the threshold was set to 0.1, more rice ears were identified, but the accuracy was low. Moreover, the background part was often identified as rice. As shown in Table 3, the experimental results of SSD have lower counting accuracy than the method in this study. In terms of labeling pictures, frame labeling is also more complicated than point labeling. In addition, the object detection algorithm requires the bounding box of each rice ear to be predicted. However, the method in this study does not need this information, thus simplifying the prediction process.

## 4. Conclusions

This study uses the case segmentation method to count rice ears in the field environment and verifies the feasibility and advancement of the LC-FCN model to count the number of rice ears. First, a rice ear dataset with a size of 3300 was produced for point-supervised research. The rice ear counting model proposed in this study uses the FCN semantic segmentation network to perform the semantic-level segmentation of rice ears. Then, the watershed algorithm was used to implement the instance segmentation of each rice ear. Finally, each rice ear was divided into a blob, and the number of rice ears was determined by counting the number of blobs. The experimental results show that compared with traditional machine learning methods and SSD object detection algorithms, the method applied in this study is both accurate and convenient. The model has an MAE of 2.99 and the accuracy rate reached 89.88%. It can calculate the number of rice ears in the field and provide reliable basic data for estimating rice yields. Future works may continue to study the environmental characteristics and characteristics of the other growth stages of rice. They may also explore more combinations of semantic segmentation networks and instances of segmentation algorithms. Further research could improve the models to enhance the accuracy of rice ear counting in practical tasks, such as yield estimation.

## Figures and Tables

**Table 1:** List of image levels, number and filter characteristics.

Level	Number	Characteristics
A	596	The color difference is relatively large, the effect is the clearest and easiest to identify.
B	935	The effect is general, the noise is large, and the distinction is obvious.
C	130	The effect is the worst, the picture is reddish and the most difficult to recognize.

**Table 2:** Data results using different backbone networks and using data augmentation methods.

Backbone	Augmentation	Dataset Size	MAE	RMSE	nRMSE (%)	Acc-Rate (%)
VGG16	/	1100	8.88	11.72	17.76	61.70
	Flip	2200	5.07	8.14	11.62	82.82
	Flip + Rotation	3300	3.96	5.93	8.72	87.48
ResNet50	/	1100	8.48	12.19	18.20	61.78
	Flip	2200	4.05	7.63	11.22	84.90
	Flip + Rotation	3300	2.99	5.40	7.01	89.88

**Table 3:** Comparison of test results with SSD and ResFCN.

Method	MAE	RMSE	Accuracy
SSD	13.62	15.79	54.14%
ResFCN	9.27	13.14	56.29%

## Data Availability

The data presented in this study are available on request from the corresponding author.
